# Mitochondrial and Oxidative Unbalance in Placentas from Mothers with SARS-CoV-2 Infection

**DOI:** 10.3390/antiox10101517

**Published:** 2021-09-24

**Authors:** Chiara Mandò, Valeria M. Savasi, Gaia M. Anelli, Silvia Corti, Anaïs Serati, Fabrizia Lisso, Chiara Tasca, Chiara Novielli, Irene Cetin

**Affiliations:** 1 Department of Biomedical and Clinical Sciences Luigi Sacco, Università degli Studi di Milano, 20157 Milano, Italy; valeria.savasi@unimi.it (V.M.S.); gaia.anelli@unimi.it (G.M.A.); anais.serati@unimi.it (A.S.); fabrizia.lisso@unimi.it (F.L.); chiara.novielli@unimi.it (C.N.); irene.cetin@unimi.it (I.C.); 2 Unit of Obstetrics and Gynecology, Luigi Sacco Hospital, ASST Fatebenefratelli-Sacco, 20157 Milano, Italy; silviacorti871@gmail.com; 3 Department of Pathophysiology and Transplantation, Università degli Studi di Milano, 20090 Milano, Italy; 4 Department of Obstetrics and Gynecology, Vittore Buzzi Hospital, ASST Fatebenefratelli-Sacco, 20154 Milano, Italy; chiaratasca92@gmail.com

**Keywords:** pregnancy, SARS-CoV-2, COVID-19, placenta, mitochondria, oxidative stress

## Abstract

SARS-CoV-2 infection has been related to adverse pregnancy outcomes. A placental role in protecting the fetus from SARS-CoV-2 infection has been documented. Nevertheless, it is still unclear how the placenta is affected in SARS-CoV-2 infection. Here we assessed placental mitochondrial (mt) and oxidative features in COVID-19 and healthy mothers. mtDNA levels, DNA oxidative damage, expression levels of genes involved in antioxidant defenses, mitochondrial dynamics and respiratory chain subunits were investigated in placentas from singleton pregnancies of 30 women with SARS-CoV-2 infection during the third trimester (12 asymptomatic, 18 symptomatic) and 16 controls. mtDNA levels decreased in COVID-19 placentas vs. controls and inversely correlated with DNA oxidative damage, which increased in the symptomatic group. Antioxidant gene expressions decreased in SARS-CoV-2 mothers (*CAT*, *GSS*). Symptomatic cases also showed a lower expression of respiratory chain (*NDUFA9*, *SDHA*, *COX4I1*) and mt dynamics (*DNM1L*, *FIS1*) genes. Alterations in placental mitochondrial features and oxidative balance in COVID-19-affected mothers might be due to the impaired intrauterine environment, generated by systemic viral effects, leading to a negative vicious circle that worsens placental oxidative stress and mitochondrial efficiency. This likely causes cell homeostasis dysregulations, raising the potential of possible long-term effects.

## 1. Introduction

The 2019 coronavirus disease (COVID-19), caused by severe acute respiratory syndrome coronavirus 2 (SARS-CoV-2), has been related to adverse pregnancy outcomes such as preterm birth, premature rupture of membranes, low birth weight and high neonatal intensive care unit admission [1,2,3,4,5]. It is well recognized that these conditions can drive negative long-term consequences, cardiovascular and metabolic diseases that can reach far into the future life of the adult [6]. Therefore, the need for a deeper understanding of in utero processes in mothers affected by COVID-19 has become urgent and essential for the proper management of these pregnancies [5].

The placenta is a metabolically active barrier that forms in utero, mediates fetal oxygenation and nutrition and protects the fetus from pathogens by activating different molecular pathways [7]. Recently, a placental role in fetal protection from SARS-CoV-2 infection has been suggested [7,8]. At the same time, evidence for vertical transmission of SARS-CoV-2 from the mother to the fetus has been documented in a small but significant percentage of cases [9,10,11]. Although histopathologic abnormalities, such as maternal vascular malperfusion (MVM), inflammation and fibrinoid deposition have been described in some studies [7,12,13,14,15], we recently showed that SARS-CoV-2 infection during the third trimester does not influence placental histological pattern when compared to appropriately matched controls [16]. It is therefore unclear how the placenta is affected in SARS-CoV-2 infection, depending also on timing and duration of exposure.

Dysfunctional placentas may lead to altered oxygenation in different pregnancy pathologies, such as IUGR [17], maternal obesity and gestational diabetes mellitus [18]. This is accompanied by altered mitochondrial features [19,20], accounting for an oxidative impairment in the placental and intrauterine environment. Indeed, mitochondria have a key role in the maintenance of cellular homeostasis and are the energy powerhouse of the cell, driving placental functions and efficiency. Interestingly, COVID-19 has been associated with acute inflammatory processes, redox imbalance, oxidative stress and altered mitochondrial dynamics [21,22,23,24]. However, to date, no data have been reported on placental tissue.

The aim of the present study was to assess the mitochondrial and oxidative features of placentas from mothers affected by COVID-19 during the third trimester of pregnancy compared to healthy control pregnancies. This will allow light to be shed on the possible role of oxidative stress and inflammatory processes characterizing SARS-CoV-2 infection on placental mitochondrial balance, with possible short- or long-term consequences.

## 2. Materials and Methods

### 2.1. Population

Pregnant women were prospectively enrolled between March and July 2020 from the Obstetrics and Gynecology Units of University Hospitals of the ASST Fatebenefratelli-Sacco in Milan. COVID cases were enrolled in the Regional Hub for COVID-19 patients at the “L. Sacco” Hospital and controls at the “V. Buzzi” Children Hospital.

The study was conducted in accordance with the Declaration of Helsinki and in compliance with all current Good Clinical Practice guidelines, local laws, regulations and organizations. The protocol was approved by the Hospital Ethical Committee (Comitato Etico Milano Area 1, protocol n° 15408, 11 March 2020). All participants gave their informed consent to collect personal data and biological samples.

Only women with singleton pregnancies who delivered at term or very late preterm (>36 gestational weeks) were included. Placentas from 30 women with a confirmed SARS-CoV-2 infection during the third trimester of pregnancy were collected and compared to 16 control healthy pregnancies. The controls were women with no maternal or fetal pathologies and normal pregnancy outcomes.

Diagnosis of maternal SARS-CoV-2 infection was performed by a positive result on a reverse transcriptase polymerase chain reaction (PCR) assay of a maternal nasopharyngeal swab specimen. The swab samples were processed with the automated ELITe InGenius system and the GeneFinder COVID-19 Plus RealAmp Kit assay, according to the manufacturer’s instructions (ELITechGroup, Inc., Bothell, WA, USA) [25]. This assay targets three genes: RNA-dependent RNA polymerase, nucleocapsid protein and envelope membrane protein, with high specificity. The test results were obtained within 24 h.

The presence of the SARS-CoV-2 infection was also evaluated in placentas, after fixation with formalin, by detecting the viral RNA by PCR. Total RNA was extracted from 3 unstained slides (5 μm thick) using Quick-RNA FFPE Miniprep (Zymo Research, Irvine, CA, USA) in an elution volume of 30 μL. The WHO/Charité SARS-CoV-2 Real-Time RT-PCR E-gene assay (Berlin, Germany) was adapted using a qPCRBIO Probe 1-Step Go Master Mix (PCR Biosystems). Human *RNase P* was used as an internal control to confirm that the RNA was adequately extracted and conserved. Positive samples were confirmed using the CE-IVD Logix Smart COVID-19 kit (Co-Diagnostic, Salt Lake City, UT, USA). According to the literature, cycle threshold values less than 40 were considered positive. The positivity to SARS-CoV-2 viral infection was established by two consecutive and positive PCR experiments.

COVID-19 cases were divided into asymptomatic (*n* = 12, with no symptoms and negative imaging when performed) and symptomatic (*n* = 18) cases. The symptomatic cases included 3 women with SaO_2_ ≤ 94%, who required respiratory support.

Maternal, obstetrical and neonatal data were collected from both cases and controls.

Neonatal weight was normalized for gestational age by calculating the *Z*-score, as previously described [26]. Briefly, for each neonate, the *Z*-score of birth weight was calculated as: [y(t) − µ(t)]/σ(t), where y(t) is the birth weight and µ(t) and σ(t) are the mean and standard deviation of the reference at gestational age, t [27].

### 2.2. Placental Collection and Molecular Analysis

Placentas were collected in sterile conditions immediately after delivery, and chorionic villi biopsies (1 cm^3^) were sampled from different sites of the placental disc (central, median and peripheral) from the maternal side. Maternal decidua was carefully peeled off the maternal side of the placenta and the villous portion of the tissue was picked up by coring 1 cm^3^ biopsies. Then the samples were washed with physiological solution to eliminate excessive blood and either immediately frozen in liquid nitrogen for mtDNA and DNA oxidative damage analysis or included in RLT lysis buffer (Qiagen, Hilden, Germany) and stored at −80 °C for gene expression analysis.

### 2.3. Mitochondrial DNA (mtDNA) Levels

Total DNA was isolated from placental tissue (~25 mg) with the spin column-based DNeasy Blood & Tissue kit (Qiagen) and its concentration measured by a NanoDrop ND-1000 spectrophotometer (NanoDrop Technologies, Wilmington, DE, USA).

mtDNA content was assessed by real-time PCR normalizing the levels of a non-polymorphic mitochondrial gene (*Cytochrome β*) to those of a single-copy nuclear gene (*RNase P*) [28]. Total DNA (30 ng) was analyzed in triplicate on the 7500 Fast Real-Time PCR system by using TaqMan chemistry (*MT-CYB*: Hs02596867_s1 and *RNase P*: 4316844; Thermo Fisher Scientific, Carlsbad, CA, USA). Cq values with standard deviation ≥ 0.25 were excluded from the post-run analysis. The mtDNA level was calculated as 2^−ΔCq^, obtained after subtracting the *RNase P* average Cq value from the *Cytochrome B* average Cq value (ΔCq).

### 2.4. DNA Oxidative Damage

The DNA oxidative damage in placental tissue was measured by the DNA/RNA Oxidative Damage (High Sensitivity) ELISA Kit (Cayman Chemical, Ann Arbor, MI, USA). This competitive ELISA covers three oxidized guanine species as markers for DNA/RNA oxidative damage: 8-hydroxy-2′-deoxyguanosine (8-OH-dG) from DNA, 8-hydroxyguanosine from RNA and 8-hydroxyguanine (8-OHG) from either DNA or RNA.

The previously isolated total DNA (specifically, 4.128 μg in Elution Buffer-AE) was denatured by heating at 97 °C for 7′, then placed on ice. Denatured DNA was digested with 2.5 units of Nuclease P1 (Product Number N8630) in ZnCl_2_ Buffer (0.1 mM, pH 5.2) (1 h 45′ at 37 °C), according to the product information sheet. Samples were then dephosphorylated by 1 unit of Alkaline Phosphatase from calf intestine (CIP enzyme, Product Number P4978) in 1X CIP Reaction Buffer (pH 7.5–8.5, 1 h at 37 °C) and inactivated with 5 mM EDTA (10′ at 75 °C). Enzymatic digestion reagents were purchased from Sigma-Aldrich (Saint Louis, MN, USA).

Treated DNA samples were then assayed for the ELISA experiment, following the manufacturer’s instructions (Product Number 589320). Samples were diluted 1:10 and analyzed in duplicate. The plate was spectrophotometrically detected at 415 nm, and an 8-point standard curve was used for concentration determination. Calculations were performed using the online spreadsheet for data analysis supplied by the manufacturer (https://www.myassays.com/8-hydroxy-2-deoxy-guanosine.assay, by Cayman Chemical, accessed on 25 September 2020).

### 2.5. Gene Expression Analysis

In 24 samples, placental tissue biopsies (~30 mg) were mechanically shredded in 600 μL of RLT Buffer (Qiagen) added with DiThioThreitol (20 uL of 2M DTT per 1 mL RLT) with a Potter homogenizer, for lysing the tissue prior to RNA isolation. Total RNA was extracted from the tissue homogenate using the column-based RNeasy Mini Kit (Product Number 74,106—Qiagen) following the manufacturer’s instructions.

RNA concentration was then quantified by NanoDrop ND-1000.

After DNase I treatment (Turbo DNase; Thermo Fisher Scientific, Vilnius, Lithuania), aiming to remove potentially contaminating DNA, total RNA was reverse transcribed by using the High Capacity cDNA Reverse Transcription Kit (Thermo Fisher Scientific) with random examers.

A triplicate cDNA analysis was performed for:

- 4 genes related to antioxidant defenses—*Catalase* (*CAT*, assay ID: Hs00156308_m1), *SuperOxide Dismutase 1* (*SOD1*, assay ID: Hs00533490_m1), *Glutathione SynthetaSe* (*GSS*, assay ID: Hs00609286_m1) and *Glutathione ReductaSe* (*GSR*, assay ID: Hs00167317_m1);
- 4 genes belonging to the respiratory chain subunits, within the inner membrane of mitochondria—*NADH-dehydrogenase-1α subcomplex 9* (*NDUFA9*, complex I; assay ID: Hs00245308_m1), *Succinate DeHydrogenAse complex subunit A* (*SDHA*, complex II; assay ID: Hs00188166_m1), *UbiQuinol-Cytochrome C Reductase core protein I* (*UQCRC1*, complex III; assay ID: Hs00163415_m1) and *Cytochrome C Oxidase subunit IV Isoform 1* (*COX4I1*, complex IV; assay ID: Hs00971639_m1);
- 3 genes related to the mitochondrial dynamics of fission and fusion—*DyNaMin-1-Like Protein 1* (*DNM1L*, assay ID: Hs01552605_m1), *mitochondrial FISsion 1 Protein* (*FIS1*, assay ID: Hs00211420_m1) and *mitochondrial dynamin-like GTPase* (*OPA1*, assay ID: Hs01047013_m1).

The relative gene expressions of the above-listed genes were determined by 7500 Fast Real-Time PCR with TaqMan assays, according to the 2^−ΔΔCq^ method [29] relative to *Ribosomal Protein L13A* (*RPL13A*, assay ID: Hs04194366_g1) selected from a pool of placental endogenous genes. Indeed, *RPL13A* shows a minimal placental site-to-site expression variability [30].

The reagents were supplied by Life Technologies (Thermo Fisher Scientific, Foster City, CA, USA).

Only Cq values with a standard deviation ≤0.25 across triplicates were included in the analysis.

### 2.6. Statistical Analysis

All data sets underwent the Kolmogorov–Smirnov test for the assessment of scores distribution.

When data presented normal distribution, the parametric one-way ANOVA with Tukey’s HSD post-hoc test was applied, with Levene’s test for homogeneity of variances. When data did not present normal distribution, the non-parametric Kruskal–Wallis test was performed, with the Mann–Whitney U test used for a post-hoc analysis when significant results were obtained.

A chi-squared test for independence with Yates Continuity Correction was applied to determine whether the results in the control and pathological samples, with or without symptoms, were related to the categorical variables: type of delivery (vaginal delivery, cesarean section), geographic origin (Caucasian, non-Caucasian), pregestational BMI (underweight, normal weight, overweight, obese), fetal sex (female, male), maternal smoking (yes/no). By using the chi-squared test, the observed frequencies of cases that occurred in each of the categories were therefore compared with the values that would have been expected if there was no association between the two variables being measured. 

The relationships between variables were investigated using the non-parametric Spearman’s Rank Order Correlation (rho).

Differences and correlations were considered significant when *p* < 0.05.

Analyses were performed using the statistical package SPSS, v.27 (IBM; Armonk, NY, USA).

## 3. Results

### 3.1. Characteristics of the Population

Table 1 shows the clinical characteristics of the population.

No significant differences were observed among the study groups, both for the continuous (one-way ANOVA) and the categorical variables (chi-squared test for independence with Yates Continuity Correction).

Table 2 reports the therapy and clinical history of the SARS-CoV-2-positive patients. The antiviral therapy was composed of Ritonavir + Lopinavir. Few patients delivered the same day they received a COVID-19 diagnosis; therefore, they did not receive a specific antepartum therapy.

Three of the thirty placentas from SARS-CoV-2-infected mothers were also positive when tested for the virus (one from an asymptomatic mother and two from symptomatic mothers).

However, the clinical and molecular data from the three COVID-19-positive placentas were similar to the average of their group.

### 3.2. Molecular Analyses

The non-parametric Kruskal–Wallis test was performed to assess the differences among the three groups (controls, asymptomatic COVID-19, symptomatic COVID-19).

#### 3.2.1. mtDNA and DNA Oxidative Damage

**Figure 1:**
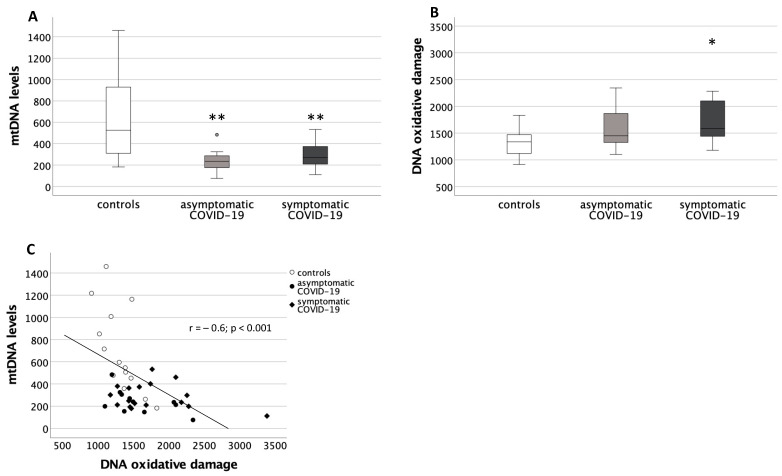
Mitochondrial DNA (mtDNA) and DNA oxidative damage levels in placentas from healthy control pregnancies and mothers infected with SARS-CoV-2 during the third trimester of pregnancy. (**A**) Placental mtDNA levels. Data are shown as box plots, indicating the median and the 25th and 75th percentiles. Data were significantly different among the three groups (Kruskal-Wallis test, *p* < 0.01). ** *p* < 0.01 vs. controls (Mann-Whitney U test), round circles are values that differ of more than 2 standard deviations from the median value. (**B**) Placental DNA oxidative damage levels. Data are shown as box plots, indicating the median and the 25th and 75th percentiles. Data were significantly different among the three groups (Kruskal-Wallis test, *p* < 0.05). * *p* < 0.05 vs. controls (Mann-Whitney U test). (**C**) Correlation between placental DNA oxidative damage and mtDNA levels. White circles: controls; black circles: asymptomatic COVID-19; black diamonds: symptomatic COVID-19.

The placental mtDNA levels were significantly different among groups (*p* < 0.01). Specifically, the Mann–Whitney U test showed significantly lower levels in the placentas from both COVID-19 groups compared to the controls (*p* < 0.01, in both cases) (Figure 1A).

The DNA oxidative damage was also significantly different among groups (*p* < 0.05), with placentas from the symptomatic COVID-19 group showing increased damage compared to the controls (*p* < 0.05) (Figure 1B).

Interestingly, a significant negative correlation was found between the placental mtDNA levels and the DNA oxidative damage levels (r = −0.6; *p* < 0.001) (Figure 1C).

#### 3.2.2. Expression of Genes Related to Oxidative Defenses

The gene expression of four antioxidant genes was measured: *CAT*, *SOD1*, *GSS* and *GSR*.

**Figure 2:**
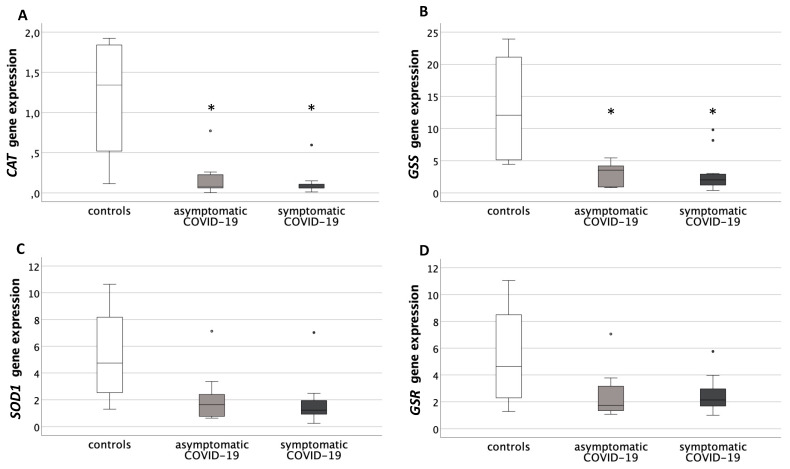
Expression levels of genes related to antioxidant defenses in placentas from healthy control pregnancies and mothers infected with SARS-CoV-2 during the third trimester of pregnancy. (**A**) Placental *Catalase* (*CAT*) gene expression levels. Data were significantly different among the three groups (Kruskal-Wallis test, *p* < 0.05). * *p* < 0.05 vs. controls (Mann-Whitney U test). (**B**) Placental *Glutathione SynthetaSe* (*GSS*) expression levels. Data were significantly different among the three groups (Kruskal-Wallis test, *p* < 0.05). * *p* < 0.05 vs. controls (Mann-Whitney U test). (**C**) Placental *SuperOxide Dismutase* (*SOD*) gene expression levels. Data were not significantly different among the three groups. (**D**) Placental *Glutathione ReductaSe* (*GSR*) gene expression levels. Data were not significantly different among the three groups. Round circles are values that differ of more than 2 standard deviations from the median value.

*CAT* placental levels were significantly different among groups (*p* < 0.05), with both COVID-19 groups showing a significant decrease in the post-hoc analysis, compared to controls (asymptomatic: *p* = 0.03; symptomatic: *p* = 0.02) (Figure 2A).

*GSS* expression was also significantly different among the three study groups (*p* < 0.05). The post-hoc analysis reported significantly decreased levels in asymptomatic COVID-19 placentas (*p* = 0.03) and in symptomatic COVID-19 placentas vs. controls (*p* = 0.02) (Figure 2B).

*SOD1* and *GSR* showed decreased levels of gene expression in the placentas of both COVID-19 groups compared to controls, though not significantly (Figure 2C,D).

#### 3.2.3. Expression of Genes Belonging to the Respiratory Chain Subunits

The gene expression of four genes belonging to the respiratory chain subunits was measured: *NDUFA9*, *SDHA*, *UQCRQ1* and *COX4I1*.

**Figure 3:**
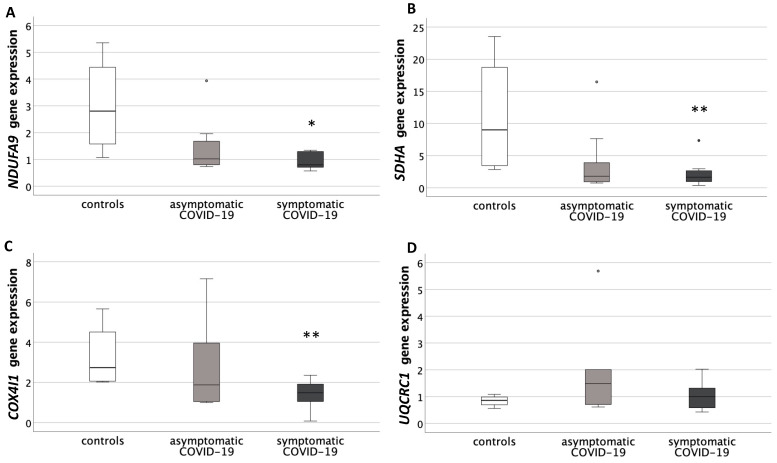
Expression levels of genes belonging to the respiratory chain subunits, within the inner membrane of mitochondria, in placentas from healthy control pregnancies and mothers infected with SARS-CoV-2 during the third trimester of pregnancy. (**A**) Placental *NADH-dehydrogenase-1α subcomplex 9* (*NDUFA9*, complex I) gene expression levels. Data were significantly different among the three groups (Kruskal–Wallis test, *p* < 0.05). * *p* < 0.05 vs. controls (Mann-Whitney U test). (**B**) Placental *Succinate DeHydrogenase complex subunit A* (*SDHA*, complex II) gene expression levels. Data were significantly different among the three groups (Kruskal-Wallis test, *p* < 0.05). ** *p* = 0.01 vs. controls (Mann-Whitney U test). (**C**) Placental *Cytochrome c OXidase subunit IV Isoform 1* (*COX4I1*, complex IV) gene expression levels. Data were not significantly different among the three groups. (**D**) Placental *UbiQuinol-Cytochrome c Reductase Core protein I* (*UQCRC1*, complex III) gene expression levels. Data were significantly different among the three groups (Kruskal–Wallis test, *p* < 0.05). ** *p* = 0.01 vs. controls (Mann-Whitney U test). Round circles are values that differ of more than 2 standard deviations from the median value.

*NDUFA9*, *SDHA* and *COX4I1* presented a significantly different gene expression among groups (*p* < 0.05, for all genes). The Mann–Whitney U test showed significantly decreased levels in symptomatic COVID-19 compared to controls (*NDUFA9*: *p* = 0.04; *SDHA*: *p* = 0.01; *COX4I1*: *p* = 0.01) (Figure 3A–C).

*UQCRC1* gene expression was not significantly different among groups (Figure 3D).

#### 3.2.4. Expression of Genes Related to Mitochondrial Dynamics of Fusion and Fission

Finally, the gene expression levels of three genes involved in the mitochondrial dynamics of fission and fusion were tested: *DNM1L*, *FIS1* and *OPA1*.

**Figure 4:**
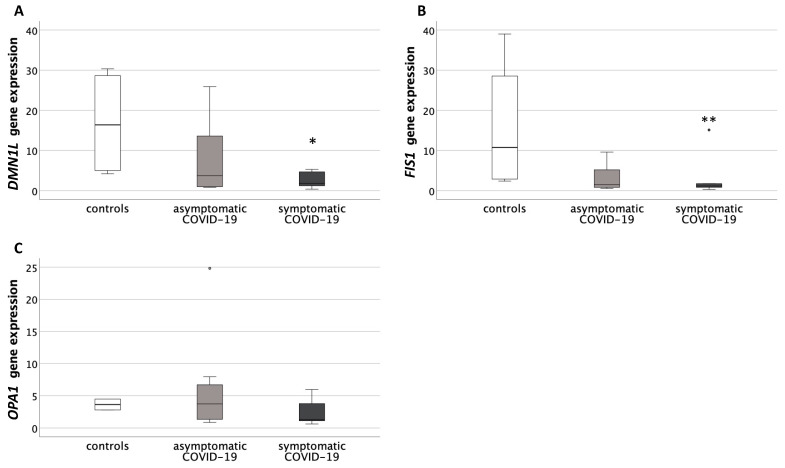
Expression levels of genes related to the mitochondrial dynamics of fusion and fission in placentas from healthy control pregnancies and mothers infected with SARS-CoV-2 during the third trimester of pregnancy. (**A**) Placental *Pro-Fission Dynamin-1-like Protein* (*DNM1L*) gene expression levels. Data were significantly different among the three groups (Kruskal-Wallis test, *p* < 0.05). * *p* < 0.05 vs. controls (Mann-Whitney U test). (**B**) Placental *Fission 1 Protein* (*FIS1*) gene expression levels. Data were significantly different among the three groups (Kruskal-Wallis test, *p* < 0.05). ** *p* = 0.01 vs. controls (Mann–Whitney U test). (**C**) Placental *Pro-Fusion mitochondrial Dynamin-like GTPase* (*OPA1*) gene expression levels. Data were not significantly different among the three groups. Round circles are values that differ of more than 2 standard deviations from the median value.

Both *DNM1L* and *FIS1* showed a significant difference in their gene expression levels among groups (*p* < 0.05, for both genes). Post-hoc analysis showed that the symptomatic COVID-19 group had significantly decreased values compared to the controls (*DNM1L*: *p* = 0.02; *FIS1*: *p* = 0.01) (Figure 4A,B).

*OPA1* gene expression was not significantly different among groups (Figure 4C).

No significant correlation was found between gestational age, pregestational BMI, gestational weight gain and maternal age in any of the analyzed molecular data.

## 4. Discussion

To our knowledge, this is the first study exploring the mitochondrial and oxidative balance at the placental level in pregnancies of mothers infected by SARS-CoV-2 during the third trimester of pregnancy. 

Alterations of placental genes and proteins involved in inflammatory and oxidative stress conditions have been previously reported in other gestational infections [31,32,33]. Interestingly, placentas of women after the exacerbation of herpesvirus infection present altered mitochondrial protein expression [34] and mitochondrial fission and fusion are dysregulated in cells affected by most RNA viruses, including Zika, hepatitis B and C, Dengue and Chikungunya [35].

Our preliminary results demonstrate that placental mtDNA levels are significantly decreased in COVID-19-infected pregnant patients, both symptomatic and asymptomatic, compared to controls. mtDNA levels were significantly and inversely related to DNA oxidative damage, a direct measure of cell oxidative stress, which was significantly increased in the most severe (symptomatic) group. Moreover, genes involved in oxidative defenses presented a significantly decreased expression in placentas of asymptomatic SARS-CoV-2-infected mothers vs. controls (namely, *CAT* and *GSS*). Similarly, COVID-19 symptomatic cases also showed significantly lower expression levels of these genes compared to controls, together with a decrease in genes belonging to the mitochondrial respiratory chain subunits (*NDUFA9*, *SDHA*, *COX4I1*) and to mitochondrial dynamics (*DNM1L*, *FIS1*).

Oxidative stress is the result of the imbalance between reactive oxygen species (ROS) and antioxidant defenses [36]. It can be generated by increased inflammation, and can also further induce inflammation, in a vicious circle exacerbating the adverse environment. Mitochondria are the major source of ROS in cells. At the same time, increased placental mitochondrial ROS may directly damage mtDNA, thus inhibiting the adaptive biogenesis of mitochondria and reducing respiratory activity [37,38,39]. This might explain our observations in the placentas of mothers infected by SARS-CoV-2 during the third trimester of pregnancy. Indeed, these placentas presented decreased antioxidant defenses and increased DNA oxidative damage, inversely related to mitochondrial DNA levels, together with decreased expression of genes belonging to the respiratory chain subunits, which also accounts for reduced mitochondrial biogenesis. Compromised antioxidant capacity coupled with oxidative stress may lead to mitochondrial damage, which in turn increases ROS production, resulting in impaired cell function and inability of compensation, thus exacerbating oxidative damage [40]. In placentas of COVID-19 mothers, the acute infection event, oxygen desaturation and placental malperfusion may therefore impede the setting up of compensation mechanisms, leading to altered mitochondrial features and redox unbalance. Similar conditions have been reported in several pregnancy pathologies. Placental oxidative stress and mitochondrial dysfunctions have been reported in a range of gestational disorders, also associated with raised inflammation, such as preeclampsia (PE), intrauterine growth restriction, gestational diabetes and maternal obesity, leading to consequences that can reach far into the future life of the adult [19,20,37,41,42,43,44,45]. In particular, we have previously reported altered mitochondrial function in preeclamptic placentas [46] and increased preeclampsia has been reported in COVID-19 patients [47]. In PE, Holland and colleagues reported decreased placental respiratory reserve capacity, with compensatory antioxidant and mitochondrial responses [48]. These findings support our hypothesis that an altered intrauterine environment characterized by enhanced inflammation and oxidative stress might lead to dysregulations of oxidative and mitochondrial features in COVID-19 placentas, with different alteration levels depending on the degree of severity. Interestingly, in non-pregnant patients, COVID-19 has been reported to provoke redox unbalance and oxidative stress, and SARS-CoV-2 infection has been associated with altered mitochondrial dynamics [21,22,23,24]. Moreover, altered circulating mitochondrial DNA has been recently reported as an indicator of severe illness and mortality in non-pregnant patients affected by COVID-19, suggesting mitochondrial dysfunction related to the disease [24].

To date, conflicting data have been reported on the macro- and microscopical morphologies of placental and fetal tissues positive to SARS-CoV-2 [15,49]. A few studies on the pregnancies of mothers affected by COVID-19 have reported increased placental inflammation and widespread abnormalities in the placental vasculature, suggesting poor perfusion and hypoxia, which may result in multiple placental malfunctions [12,13,50,51]. However, most of these studies lacked control groups, allowing limited conclusions on placental findings following maternal SARS-CoV-2 infection. Indeed, other studies did not account for fetal or maternal vascular malperfusion [16,52]. In a recent prospective multicenter case-control study, we reported no differences in the placental histological pattern in a cohort of mothers affected by SARS-CoV-2 at the end of pregnancy compared to controls with similar maternal characteristics [16]. In these cases, most of the infected women were asymptomatic and the duration of the insult had been likely too short to induce histological alterations. This cannot exclude molecular alterations in mitochondrial and oxidative pathways, due to the increased inflammatory environment reported in mothers positive to SARS-CoV-2. Indeed, we also reported evidence of increased immune activation profiles in SARS-CoV-2-positive subjects, with higher levels of cytokines and chemokines in placental tissue and maternal and funicular plasma [9].

In the symptomatic COVID-19 population, impaired mitochondrial dynamics were also found, with significantly decreased gene expressions of *DNM1L* and *FIS1*, both involved in mitochondrial fission, counterposed to no differences in the pro-fusion *OPA1* expression. This is consistent with results on mtDNA and respiratory chain subunits expression, as reduced mitochondrial fission can lead to a decrease in the formation of healthy mitochondria [37]. In general, an overall shift toward less fission and more fusion events has been associated with cell senescence, higher ROS production and decreased mitochondrial activity [53,54]. Interestingly, Parone and colleagues reported that preventing mitochondrial fission in mammalian cells leads to a loss of mitochondrial DNA, impaired mitochondrial function and increased ROS levels, suggesting that mitochondrial fission is necessary for the maintenance of mitochondrial function and thereby to upkeep cellular homeostasis [55]. On the other hand, an increase in *OPA1* has been suggested as a compensatory mechanism for increased mitochondrial content in preeclampsia, potentially by the stabilization of mitochondrial structures [56]. Indeed, overexpression of *OPA1* protects from heart and brain ischemia and from ROS production [57]. 

### Strengths and Limitations of the Study

All placentas analyzed in the present study were collected during the first period of the COVID-19 pandemic, between March and July 2020, when the pandemic’s management and hospital organization were still being set up in Italy and all around the world. Nevertheless, the dedication of the doctors and midwives to this research project allowed the sample collection immediately after delivery. Moreover, the research group dedicated to the sample analysis was located very close to the delivery and surgery rooms, thus allowing immediate processing and proper conservation of samples.

Another strength of this work is represented by the well-characterized study population, which was composed of women with similar clinical characteristics in the cases and controls.

However, although we did not observe any differences between the cases and controls in the maternal pregestational BMI and other maternal characteristics, we cannot exclude that part of the reported differences between the infected and non-infected women may be due to the intrinsic characteristics of the population. Indeed, we previously reported that pregestational BMI can contribute to the severity of the disease, representing an inflammatory susceptibility factor [24]. However, only three severe cases were included in the present study, which suggests that alterations in the placental oxidative balance can also occur in non-severe, non-obese patients. Pharmacological treatments might also have impacted the placental inflammatory and oxidative features. Nevertheless, none of patients infected with SARS-CoV-2 in our population had received a cortisone therapy or specific anti-inflammatory treatments.

The investigation of mitochondrial and oxidative parameters in a larger population is also mandatory to draw any final conclusions. However, despite the low number of cases, this work lays the foundations for a deeper understanding of intrauterine processes occurring in mothers affected by COVID-19. 

## 5. Conclusions

Altogether, our results suggest that changes in the placental mitochondrial features and oxidative balance in mothers infected by SARS-CoV-2 might be the result of an impaired intrauterine environment generated by the systemic viral effects, leading to a negative vicious circle involving COVID-19-enhanced systemic inflammation, which worsens placental oxidative stress and mitochondrial efficiency. The reported placental alterations are likely to make this fundamental organ more vulnerable and to provoke alterations in cell homeostasis, thereby raising the potential of further insults and possible long-term effects.

## Figures and Tables

**Table 1:** Clinical characteristics of the population. Values are presented as mean ± standard deviation. BMI: Body mass index; CS: cesarean section; UA: umbilical artery.

	Controls (*n* = 16)	Asymptomatic COVID-19 (*n* = 12)	Symptomatic COVID-19 (*n* = 18)
Pregestational BMI (Kg/m^2^)	23.2 ± 3.4	23.8 ± 5.1	25.4 ± 4.5
Gestational weight gain (Kg)	12.8 ± 3.5	13.2 ± 5.3	11.1 ± 4.1
Maternal age (years)	32.9 ± 4.9	32.2 ± 3.8	31.8 ± 6.0
Maternal hematocrit	33.7 ± 3.1	34.5 ± 2.4	33.9 ± 3.1
Maternal hemoglobin (mg/dL)	11.4 ± 1.2	11.7 ± 1.0	11.4 ± 1.5
Maternal geographic origin (% Caucasian)	69%	80%	63%
Maternal smoking (n. of smokers)	0	0	1
Mode of delivery (% CS)	31%	30%	26%
Gestational age (weeks)	39.9 ± 1.1	39.1 ± 1.4	38.9 ± 1.4
Birth weight (g)	3487 ± 298	3257 ± 416	3239 ± 395
Birth weight (*Z*-score)	0.24 ± 0.81	−0.05 ± 1.15	0.015 ± 1.17
Fetal sex (% males)	50.0%	30.0%	47.4%
UA pH	7.30 ± 0.09	7.36 ± 0.08	7.32 ± 0.08

**Table 2:** Therapy and clinical history of the SARS-CoV-2-positive patients. NPS: nasopharyngeal swab. SD: standard deviation.

	Asymptomatic COVID-19 (*n* = 12)	Symptomatic COVID-19 (*n* = 18)
***Antepartum therapy***		
Antiviral (%)	8%	44%
Oxygen support (%)	0%	22%
Days between a positive NPS and delivery (mean ± SD)	1.5 ± 3.4	5.4 ± 9.6
***Vital signs***		
Fever ≥ 37 °C (%)	0%	44%
Respiratory rate ≥ 20 (%)	8%	39%
Heart rate ≥ 100 bpm (%)	0%	22%

## Data Availability

The data are contained within the article.
